# Tannerella forsythia is associated with increased levels of atherogenic low density lipoprotein and total cholesterol in chronic periodontitis

**DOI:** 10.4317/jced.52128

**Published:** 2015-04-01

**Authors:** Carlos M. Ardila, Aide-Yancelly Perez-Valencia, Willer-Leandro Rendon-Osorio

**Affiliations:** 1 Periodontist. Ph.D in Epidemiology, Biomedical Stomatology Group, Universidad de Antioquia U de A, Medellín, Colombia, Department of Periodontology, School of Dentistry, Universidad de Antioquia; 2 Dentist, Department of Periodontology, School of Dentistry, Universidad de Antioquia

## Abstract

**Background:**

Accumulating evidence suggests that acute and chronic infections with periodontopathogens are associated with an increased risk of cardiovascular disease. The objective of this study was to assess whether Tanerella forsythia and Porphyromonas gingivalis are associated with increased levels of atherogenic low-density lipoprotein (LDL), high-density lipoprotein, total cholesterol (TC), triglycerides and body mass index (BMI) in chronic periodontitis patients.

**Material and Methods:**

Medical history and clinical and radiographic examination were conducted in 80 chronic periodontitis patients and 28 healthy individuals. Fasting blood samples were drawn for the measurement of the parameters of dyslipidemia. Anthropometric measurements such as height in meters and weight in kilograms were recorded. Both periodontitis and control subjects were asked to answer a questionnaire with regard to their socio-demographic and smoking status. The presence of T. forsythia, and P. gingivalis was detected using primers designed to target the respective 16S rRNA gene sequences.

**Results:**

The occurrence of T. forsythia and P. gingivalis was higher in the group of subjects with periodontitis. Superior levels of triglycerides were observed in chronic periodontitis patients compared to healthy individuals. High levels of TC in periodontitis persons were significantly associated with increased bleeding on probing. Greater mean levels of TC and LDL were shown in the presence of T. forsythia (P<0.05). Likewise, higher proportions of patients with BMI ≥25 kg/m2 related with T. forsythia (P<0.05). T. forsythia was a significant discriminating factor in the multivariate linear regression model emerging as significant explanatory of increased levels of TC (β=17,879, 95% CI = 4,357-31,401; p=0.01) and LDL (β=17,162, 95% CI= 4,009-30,316; p=0.01).

**Conclusions:**

Higher levels of serum total cholesterol and LDL were observed in the occurrence of T. forsythia and the presence of this periodontopathogen may increase the atherogenic potency of low-density lipoprotein that may augment the risk for atherosclerosis in periodontal disease patients.

** Key words:**Periodontitis, dyslipidemia, Tannerella forsythia, cardiovascular disease.

## Introduction

Dyslipidemia is recognized as one of the major modifiable risk factors for coronary heart disease. Nevertheless, some patients with atherosclerosis lack presently of recognized risk factors including hypertension, hypercholesterolemia, diabetes and smoking, suggesting the occurrence of other causative mechanisms (1). Thus, infectious agents signify a main resource of systemic inflammatory reaction with the possibility to step up plaque growth and instability (2). Accumulating evidence suggests that acute and chronic infections with periodontopathogens are associated with an increased risk of cardiovascular disease (3). The susceptibility of the arterial wall to bacterial infection is increased in the existence of other cardiovascular risk factors, such as dyslipidemia, suggesting that chronic infection may incline an individual to atherosclerosis (4).

Among the microorganisms observed in atherosclerotic vessels, *Porphyromonas gingivalis* does not emerge leading and it is infrequently detected without the occurrence of other organisms (5), which highlights the magnitude of studying how polymicrobial infection influences atherosclerosis development. *Tannerella forsythia* has remained an under investigated organism because of its fastidious growth and recalcitrant nature to genetic manipulation (6). Similarly to *P. gingivalis*, *T. forsythia* has been detected in human clinical atherosclerotic plaque lesions (7). Therefore, it is also important to study the relationship of the presence of these two microorganisms with dyslipidemia. Previous investigations showed that periodontitis is associated with increased levels of low-density lipoprotein (LDL), total cholesterol (TC), and triglycerides (TGs) (8,9). Similarly, as pointed out by Tu *et al.* (10), periodontitis patients had higher levels of body mass index (BMI) compared to healthy controls. Conversely, it was shown that periodontitis decreases the level of high-density lipoprotein (HDL) (10-13).

To our knowledge no studies have looked the influence of *T. forsythia* on the levels of LDL, HDL, TC, TGs and BMI. Thus, the objective of this study was to assess whether *T. forsythia* and *P. gingivalis* are associated with augmented levels of atherogenic LDL, HDL, total cholesterol, triglycerides and body mass index.

## Material and Methods

-Patient selection

Patients with a diagnosis of chronic periodontitis were considered candidates for the study. A total of 79 women and 29 men (aged 33 to 82 years) with ≥ 18 residual teeth who attended the dental clinics of the Universidad de Antioquia in Medellín, Colombia were invited to participate in this study between January 2009 and December 2011. Of the 108 subjects included, 28 patients belonged to the control group (subjects without periodontitis).

The study design was approved by the Ethics Committee on Human Research of the School of Dentistry of the University of Antioquia according to the Declaration of Helsinki on experimentation involving human subjects. Informed and written consent was obtained from each participant. Exclusion criteria included diagnosed diabetes and autoimmune diseases. Pregnant women, previous (six months) consumption of systemic antimicrobials, non-steroidal analgesics or anti-inflammatory drugs, and previous periodontal therapy also served as exclusion criteria.

-Clinical Evaluation

Clinical and radiographic examination and medical history were conducted for each person. The diagnosis of chronic periodontitis was made based on criteria defined by Eke *et al.* (14); subjects were classified as moderate periodontitis by ≥2 interproximal sites with clinical attachment level (CAL) ≥4 mm, or by ≥2 interproximal sites with probing depth (PD) ≥5 mm (not at the same tooth). Severe periodontitis was characterized by ≥2 interproximal sites with CAL ≥6 mm and ≥1 inter-proximal site with PD ≥5 mm (not at the same tooth). Subjects with no evidence of mild, moderate, or severe periodontitis were used as a control group.

A trained and calibrated clinician performed all clinical examinations. The intra-examiner reproducibility was assessed before and during the study. The intra-class correlation coefficients for mean PD and CAL was 0.92 and 0.91, respectively; the intra-evaluator kappa index was in the range 0.85-0.96. The presence or absence of bleeding on probing (BOP) and plaque were registered. PD and CAL were measured at all proximal, buccal and lingual surfaces to the nearest millimeter by a calibrated standard probe (UNC-15, Hu-Friedy, Chicago, IL).

The presence or absence of plaque was registered qualitatively. If bleeding occurred immediately after probing for pocket depth it was reported as positive. Probing depth is the distance between the gingival margin and to the bottom of the gingival pocket measured from six angles of each tooth. Gingival pockets 4 mm or deeper were considered to be pathogenic. CAL was determined at all sites by measuring the distance from the cemento-enamel junction (CEJ) to the free gingival margin (GM), adding the PD at the same site. CAL = PD + (CEJ to GM) (all measurements in millimeters).

-Medical Examination

Fasting blood samples were drawn for the measurement of hemoglobin A1c (HbA1c), TC, HDL, LDL and TGs. The diagnosis of adipose tissue disorders was made according to the criteria defined by the Third Adult Treatment Panel of the National Cholesterol Education Program (15). To identify subjects with pathological values the following cut-off points were used: total cholesterol ≥ 200 mg/dl; HDL<40 mg/dl in men and <50 mg/dl in women, respectively; low density lipoproteins (LDL) ≥130 mg/dl; serum triglycerides > 150 mg/dl); and body mass index (≥25 kg/m2). Serum total cholesterol, HDL cholesterol, and triglyceride concentrations were determined by fully enzymatic methods in the same local laboratory of clinical chemistry. These values are applica-ble to individual with a normal risk for cardiovascular disease (15).

Anthropometric measurements such as height in meters and weight in kilograms were recorded. Body mass index was calculated using the formula BMI= Weight/Height (15).

Both periodontitis and control subjects were asked to answer a questionnaire with regard to their socio demographic and smoking status including gender (male/female), age (in years), smoking status (smoker/non smoker) and socioeconomic status (US≥750 income per month; yes/no).

-Microbial Sampling

Microbial sampling on periodontitis patients was performed on pockets > 5 mm. The deepest six pockets were selected for sampling. After removing supragingival plaque with curette and isolating the area with cotton pellets, the paper points (Maillefer, Ballaigues, Switzerland) were inserted into each periodontal pocket for 20 seconds. One paper point from each site was introduced into an empty 1.5 ml micro-fuge tube for polymerase chain reaction (PCR) analysis.

The presence of *T. forsythia*, and *P. gingivalis* was detected by PCR using primers designed to target the respective 16S rRNA gene sequences, according to the method of Ashimoto *et al.* (16). Briefly, PCR mixtures (50 mL) were prepared with 5 mL of bacterial DNA (GoTaq Flexi DNA Polymerase, Promega), 0.5 mM species-specific primers, 10 mL of 5 PCR buffer (Deoxynu-cleotide Triphosphates, Promega), 1.25 U of Taq DNA polymerase (Deoxynucleotide Triphosphates, Promega), 0.2 mMdNTP mix (Deoxynucleotide Triphosphates, Promega), and 1.5 mM MgCl2 (Promega). Gene-specific amplification was performed in a thermal cycler (MyCycler® Termal Cycler, Bio-Rad) with the following thermal profiles: *T. forsythia* and *P. gingivalis*, initial denaturation step at 95°C for 2 minutes, followed by 36 cycles of denaturation at 95°C for 30 seconds, annealing at 60°C for 1 minute and extension at 72°C for 1 minute, and a final extension at 72°C for 2 minutes. PCR products were electrophoresed on 1% agarose gels and stained with 0.5 mg/mL ethidium bromide, and the presence of target bands for each bacterium was confirmed.

-Sample size calculation

The sample size was calculated with a power of 80% and a significance level of 0.05 (two-tailed) for detecting a risk ratio ≥ 2 for a prevalence of TGs > 150 mg/dl of 70.83% and 29.17% (based on a previous study) (17) in the groups with and without periodontitis, respectively. Based on these calculations, it was determined that ≥ 22 persons per group would be necessary.

-Statistical Analysis

Differences in continuous and categorical variables were examined with independent t test (data were distributed normally) and X2 test, respectively. Linear regression analysis was applied stepwise, and two final models were constructed for the dependent variable TC (mg/dl) and LDL (mg/dl), respectively. Variables included in the model were age (years), gender (male/female), smoking status (smoker/non smoker), socioeconomic status (US≥750 income per month; yes/no), presence of *P. gingivalis* (yes/no), and *T. forsythia* (yes/no). Multicollinearity was not found during model fitting. Regression coefficients, standard errors, and adjusted R2 were expressed.

Besides, associations between BMI (<25 kg/m2 versus ≥25 kg/m2) and presence of *P. gingivalis*, and *T. forsythia* were assessed by logistic regression analysis. The odds ratio (OR) and corresponding 95% confidence intervals (CI) was calculated. *P* values of <0.05 were considered statistically significant. All analyses were performed using statistical software (SPSS version 18.0; SPSS, Chicago, IL).

## Results

Eighteen women and ten men without periodontitis and 61 women and 19 men with chronic periodontitis were studied. The socio-demographic characteristics, periodontal parameters, body mass index and serum parameters of the patients with and without periodontitis are described in the  Table 1. The socio-demographic variables did not show statistically significant differences between the two groups, which make them comparable.

**Table 1:**
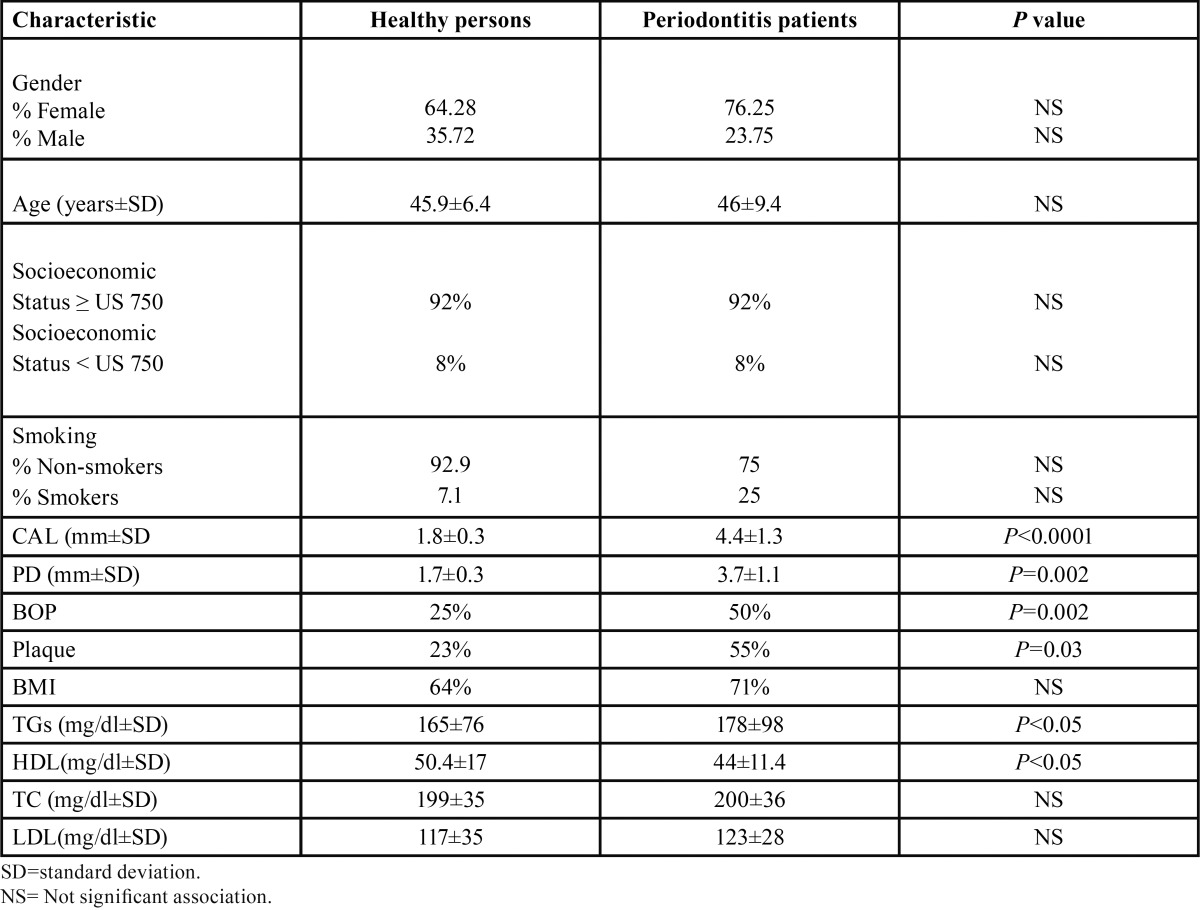
Socio-demographic features, periodontal parameters, body mass index and lipids characters of the studied patients.

There was a significant difference between both groups in relation to periodontal parameters. Similarly, the levels of TGs were higher in periodontitis patients compared to individuals without periodontitis ( Table 1).

On the other hand, the proportion of occurrence of *T. forsythia* (57.5% versus 28.5%; *P*=0.008) and *P. gingivalis* (76.2% versus 10.7%; *P*<0.0001) was higher in the group of patients with periodontitis.

 Table 2 depicts the occurrence of pathologic lipid levels in the study subjects. Except for LDL, there were significant differences between both groups. An important finding of our work was that high levels of TC in periodontitis persons were significantly associated with increased BOP (*P*<0.05).

**Table 2:**
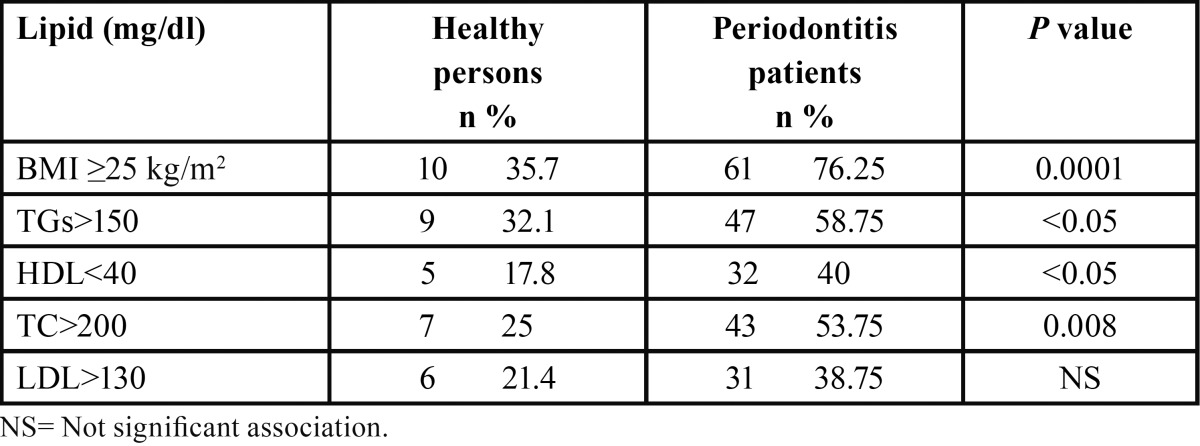
Presence of blood lipids concentrations and BMI 25Kg/m2 in the patients evaluated.

With this information, we studied whether the presence of *T. forsythia* or *P. gingivalis* correlated with high mean levels of TC, TGs, HDL and LDL, using an independent t test. Higher mean levels of TC and LDL were shown in presence of *T. forsythia* (*P*<0.05) ( Table 3). Likewise, higher proportions of patients with BMI ≥25 kg/m2 related with *T. forsythia* (*P*<0.05). Conversely, patients with or without presence of *P. gingivalis* did not present differences in the mean levels of the parameters of dyslipidemia.

**Table 3:**
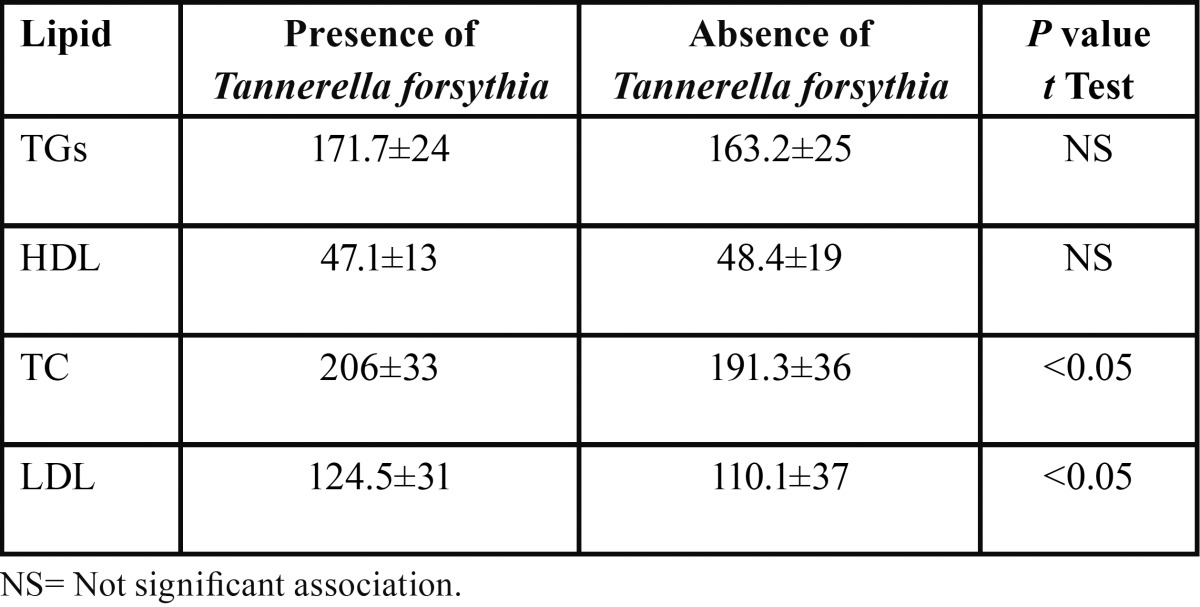
Blood lipid levels (mg/dl; mean ± standard deviation) in patients with or without *Tannerella forsythia*.

Thus, we analyzed whether the presence of *T. forsythia* is a risk factor for high levels of TC, LDL, using a linear regression model adjusted for age, gender, smoking and socioeconomic status. Likewise, whether the presence of *T. forsythia* is a risk factor for overweight was investigated using a logistic regression model adjusted for age, gender, BMI, smoking and socioeconomic status.

 Table 4 depicts the linear models. Thus, in the simple linear model, the presence of *T. forsythia* was significantly associated with high levels of TC. In the multivariate model, this statistically significant association remained after adjustment for possible confounders. Similarly, in the simple linear model, the presence of *T. forsythia* was significantly associated with high levels of LDL. This statistically significant association also remained after adjustment for possible confounders. By use of the multivariate linear regression model, we wanted to identify explanatory factors for a difference in TC and LDL. *T. forsythia* came out as significant discriminating factor in the model. Therefore, *T. forsythia* emerged as significant explanatory of increased levels of TC and LDL.

**Table 4:**
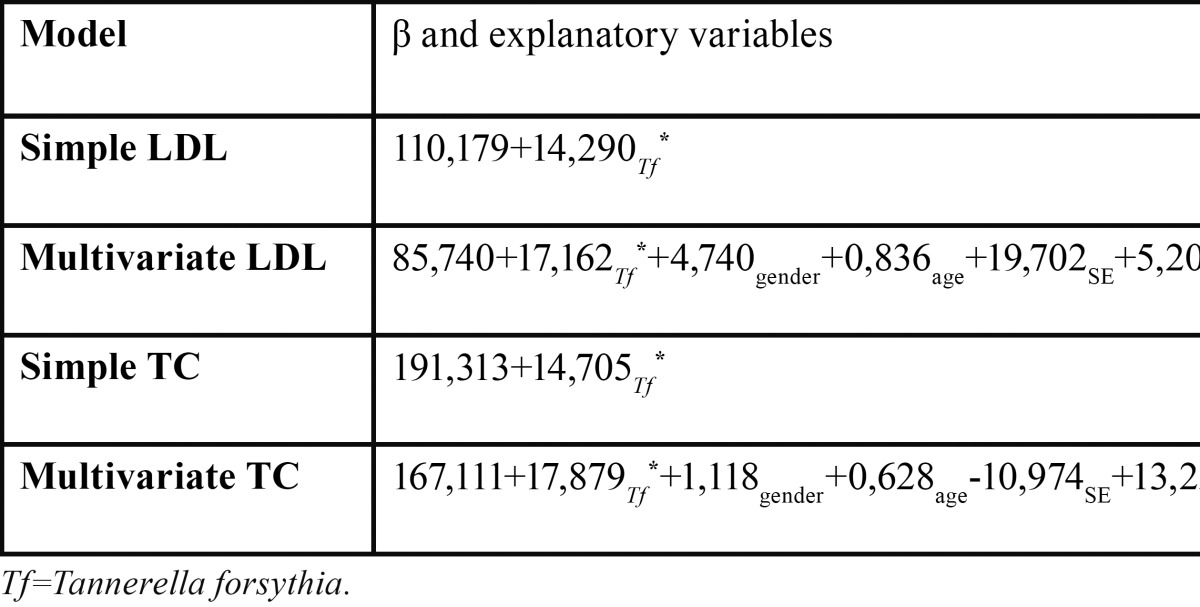
Simple and Multivariate linear regression models for LDL and TC.

On the other hand, in the crude logistic model, the presence of *T. forsythia* was not associated with overweight (OR= 1.5, 95% CI= 0.9-2.5).

## Discussion

To our knowledge, the current investigation is the first to explore the relationship of *T. forsythia* on the levels of LDL and TC, offering supplementary evidence for the relation between periodontitis and atherosclerosis, discernable by high levels of LDL and TC in the presence of *T. forsythia*.

A conceivable method accounting for the described association between periodontitis and cardiovascular disease could be the release of bacteria, bacterial products or pro-inflammatory cytokines from the chronic periodontal lesion into the blood stream (11).

T. forsythia is frequently located cohabiting with *P. gingivalis* in subgingival dental biofilms (18), which also exerts a potential impact on systemic health. T. forsythia lipopolysaccharide (LPS) could be associated with the up-regulation of pro-inflammatory cytokines and it was revealed to stimulate their production by human macrophages, with the IL-8 secretion level of *T. forsythia* LPS being about 1.5 times the effect of *P. gingivalis* LPS (19). Besides, *T. forsythia* and BspA (its major surface virulence factor) augmented atherosclerotic lesion progression in ApoE-/- mice. This process may be associated with down regulation of lipid metabolism-related gene expression (20).

In the current study, robust associations between the presence of *T. forsythia* and high LDL and TC mean levels in periodontitis subjects were observed. These results are very significant since LDL is the major atherogenic lipoprotein and TC is a crude surrogate for LDL in risk assessment or in estimating early response to treatment, however it can be convenient in primary detection or long-term monitoring of responding (21). Notably, the above aspect shows the consistency of our results. Furthermore and consistently with this relevant finding, the occurrences of LDL and TC were higher in periodontitis patients compared to individuals without periodontitis ( Table 2); similarly the mean levels also were higher in the presence of *T. forsythia* ( Table 3). Moreover, the levels of TGs were greater in periodontitis patients compared to persons without periodontitis (*P*<0.05). Augmentation of plasma triglycerides has been perceived principally in infection with gram-negative microorganism and these modifications are expedited by cytokines, which may be formed in the inflamed periodontal tissue (22). Our results are coherent with the conclusions of earlier studies (11,12). Therefore, these results corroborate the proposition that chronic infections involving periodontitis may modify the serum lipid profile in a manner that raises the risk of atherosclerosis (8).

LDL levels were detected in 31 of 80 chronic periodontitis patients, and in 6 of 28 persons without periodontitis. Buhlin *et al.* (11) presented equivalent conclusions. A possible explanation for these effects could be related to Gram-negative bacteria and free LPS in the plasma originating a release of inflammatory mediators (11,23).

As was described previously, high LDL and TC levels in periodontitis patients with occurrence of *T. forsythia* were detected, but this was not the situation for periodontitis patients with presence of *P. gingivalis*. This contrast may be due to the particular pathogenic characters of the two periodontopathogens, particularly by specific LPS and BspA in *T. forsythia* (24,25).

One additional significant conclusion of this study was that high levels of TC in periodontitis patients were significantly associated with augmented BOP (*P*<0.05). A previous study also suggested an association between dyslipidemia and BOP (11). Comparable conclusions have been defined for the relationship gum bleeding and higher LDL cholesterol (26). Longitudinal epidemiological studies with larger and more varied samples are needed to further evaluate the relationship between periodontitis and dyslipidemia.

A limitation of this investigation is its cross-sectional approach, nevertheless, an association was observed between high mean levels of TC and LDL in the occurrence of *T. forsythia* in periodontitis patients, after adjusting for various potential confounders.

## Conclusions

This investigation offers additional evidence for the association between periodontitis and atherosclerosis, marked by higher levels of TC and LDL in the presence of *T. forsythia*. These findings propose that the occurrence of *T. forsythia* increases the atherogenic potency of LDL and may intensify the risk for cardiovascular disease.
